# Quality of life among older informal caregivers in Sweden: the role of loneliness and social isolation

**DOI:** 10.1007/s11136-025-04156-x

**Published:** 2026-02-01

**Authors:** Mariam Kirvalidze, Maider Mateo-Abad, Giorgi Beridze, Amaya Bernal-Alonso, Maria João Forjaz, Carmen Rodríguez-Blázquez, Amaia Calderón-Larrañaga

**Affiliations:** 1 Aging Research Center, Department of Neurobiology, Care Sciences and Society, Karolinska Institutet and Stockholm University, Solna, Sweden; 2 Biogipuzkoa Health Research Institute, San Sebastián, Spain; 3 Research Network on Chronicity, Primary Care and Prevention and Health Promotion (RICAPPS), Girona, Spain; 4 National School of Health, Carlos III Institute of Health, Madrid, Spain; 5 National Centre of Epidemiology, Carlos III Institute of Health, Madrid, Spain; 6 Network Center for Biomedical Research in Neurodegenerative Diseases (CIBERNED), Madrid, Spain; 7 Stockholm Gerontology Research Center, Solna, Sweden

**Keywords:** Mental health, Informal care, Loneliness, Caregiving, Quality of life, Older adults

## Abstract

**Purpose:**

Older adults are increasingly taking up caregiving roles due to the mismatch between available formal care services and growing demands. We aimed to identify profiles of older caregivers according to their quality of life (QoL), and to explore the associations of such profiles with loneliness and social isolation.

**Methods:**

A cross-sectional analysis was conducted using cohort data from the Swedish National study on Aging and Care in Kungsholmen (SNAC-K). The study included a total of 994 unique caregivers aged 60 and above, assessed between 2001 and 2016. Multiple correspondence analysis and cluster analysis were used to obtain caregiver profiles according to the items of SF-12 QoL instrument. Multinomial logistic regressions with robust standard errors were performed to study the associations between QoL, loneliness and social isolation.

**Results:**

Three distinct QoL profiles were identified: *good* (57.9%), *moderate* (34.8%), and *moderate physical*,* poor mental* (7.3%) QoL. The latter profile was characterized by the predominance of female spousal caregivers, who provided the most hours of care. Loneliness and social isolation were independently associated with higher odds of being in the *moderate physical*,* poor mental QoL* profile, compared to the *good QoL* profile. Men with higher social isolation levels were more likely to be in the worse QoL profile compared to women with similarly high levels of isolation.

**Conclusion:**

Our findings emphasize that a substantial proportion of older caregivers have suboptimal QoL, and that those with poor mental QoL also experience higher levels of loneliness and social isolation. Targeted policies to reduce caregiver burden and enhance their QoL are essential.

**Supplementary Information:**

The online version contains supplementary material available at 10.1007/s11136-025-04156-x.

## Abstract (plain language summary)

Older adults are increasingly taking on caregiving roles due to gaps in formal care services. This study examines how loneliness and social isolation correlate with the quality of life (QoL) among older caregivers, identifying distinct profiles within this group. Using data from 994 caregivers aged 60 and older in the Swedish National study on Aging and Care in Kungsholmen (SNAC-K), researchers classified caregivers into three QoL profiles: *good *(57.9%), *moderate *(34.8%), and *moderate physical, poor mental QoL* (7.3%).

Caregivers in the poorest mental health profile were predominantly female spouses providing the most hours of care. Loneliness and social isolation were strongly associated with being in this group compared to those with good QoL. Additionally, men with higher levels of social isolation were more likely than women with similarly high levels of isolation to experience worse QoL, suggesting a gendered impact of social isolation on QoL.

These findings reveal that many older caregivers face significant mental and social challenges, particularly those providing intensive care or experiencing high isolation. The study emphasizes the urgent need for targeted policies to alleviate caregiver burden and improve their QoL through support programs addressing mental health, loneliness, and social connections.

**Supplementary Information:**

The online version contains supplementary material available at 10.1007/s11136-025-04156-x.

## Plain english summary

Older adults are increasingly taking on caregiving roles due to gaps in formal care services. This study examines how loneliness and social isolation correlate with the quality of life (QoL) among older caregivers, identifying distinct profiles within this group. Using data from 994 caregivers aged 60 and older in the Swedish National study on Aging and Care in Kungsholmen (SNAC-K), researchers classified caregivers into three QoL profiles: *good *(57.9%), *moderate *(34.8%), and *moderate physical, poor mental QoL* (7.3%).

Caregivers in the poorest mental health profile were predominantly female spouses providing the most hours of care. Loneliness and social isolation were strongly associated with being in this group compared to those with good QoL. Additionally, men with higher levels of social isolation were more likely than women with similarly high levels of isolation to experience worse QoL, suggesting a gendered impact of social isolation on QoL.

These findings reveal that many older caregivers face significant mental and social challenges, particularly those providing intensive care or experiencing high isolation. The study emphasizes the urgent need for targeted policies to alleviate caregiver burden and improve their QoL through support programs addressing mental health, loneliness, and social connections.

## Introduction

The demand for medical and social care is rising due to aging populations worldwide, driven by declining fertility rates [1] and increasing life expectancy, especially among the oldest old [2]. In Sweden, while the welfare system provides substantial support through home care and residential care for people in need, these services are becoming increasingly insufficient [3–5]. As a result, informal caregivers are taking on a significant share of the caregiving activities, often stepping in where formal care falls short.

In light of the increased prevalence of informal caregiving, its impact on caregivers’ lives has been a topic of interest. While some research suggests that the physical and mental demands of caregiving can negatively impact caregivers’ well-being, leading to heightened stress, anxiety, and physical illness [6–9], other studies have identified potential positive outcomes associated with caregiving, such as a sense of purpose and fulfillment [10–12]. Recently, increased attention has been devoted to social isolation (objective lack of social contacts) and loneliness (subjective feelings of being lonely) among caregivers. Most studies report increased loneliness, particularly among spousal caregivers [13, 14], but some suggest that caregiving may reduce caregivers’ loneliness by fostering meaningful connections [15]. In addition, previous studies have shown that loneliness and social isolation are not highly correlated (*r* = 0.201 [16]), and predict health outcomes through distinct pathways [17], warranting their consideration as separate, albeit connected, entities.

There is limited evidence on the impact of caregiving on older informal caregivers, who often face their own age-related health challenges and poorer well-being. Understanding their specific needs is thus essential for developing tailored support services. One way well-being has been operationalized is through quality of life (QoL), a concept that encompasses both physical and mental aspects of well-being. QoL measures are known to predict health outcomes, healthcare utilization, and the effectiveness of interventions [18].

This study aims to: (1) identify and describe profiles of older informal caregivers (60 years and above) in Sweden based on their QoL, and (2) explore the relationships between loneliness, social isolation and the different QoL profiles.

## Methods

### Study design

A completed STrengthening the Reporting of OBservational studies in Epidemiology (STROBE) checklist is available in the supplementary materials (Supplementary Table 1). This study employed a cross-sectional analysis of data from the Swedish National study on Aging and Care in Kungsholmen (SNAC-K, https://www.snac-k.se/). Each SNAC-K wave was treated as a separate observation, and we accounted for potential within-individual correlation by using robust standard errors for clustered data (see below). The study includes participants above the age of 60 from waves 1 (2001–2004), 2 (2004–2007), 3 (2007–2010), 4 (2010–2013) and 5 (2013–2016). SNAC-K assessments and interviews take place every three years for older cohorts (78+) and every six years for younger cohorts (60–78), and include examinations by a physician, psychologist, and nurse, as well as the administration of extensive self-reported questionnaires. The sample of caregivers was comprised of individuals responding “yes” to a question about whether they were providing informal care in any of the five waves.

## Study variables

The primary outcome assessed was QoL, measured using the items from the SF-12 instrument modified for the SNAC-K population study, which includes 11 of the 12 original items (missing item 7: “Did work or activities less carefully than usual”). SF-12 is a widely used tool, comprising six physical health-related (items 1, 2, 3, 4, 5, 8, amounting to 20 points) and six mental-health related (items 6, 7, 9, 10, 11, 12, amounting to 25 points) questions [19]. We used 11 items available in SNAC-K and derived distinct profiles of caregivers based on their responses, using data-driven techniques described below. To describe caregiver QoL profiles, sociodemographic characteristics such as sex, age, education level (elementary, high school, university), occupation (manual, non-manual worker), and marital status (unmarried, married, divorced, widow) were used. To approximate socioeconomic position, education was included instead of income because it is a stable indicator across the life course and remains meaningful after retirement [20]. Lifestyle factors such as smoking (never, former, current), alcohol consumption (never/occasionally, light-moderate, heavy), body mass index (underweight, normal weight, overweight, obese), and physical activity level (inadequate, health-enhancing, fitness-enhancing) were also considered using previous operationalizations in SNAC-K [21]. Health status was assessed through three indicators. Namely, the Health Assessment Tool [22], which takes values from 0 to 10 (higher scores indicating better health), the Montgomery-Åsberg Depression Rating Scale (range 0–60, with higher scores indicating more depressive symptoms), and a variable indicating whether caregivers received informal/formal care for their own needs. Caregiving-specific variables included: hours of care provided in the last month, categorized into three groups (<1h, 10% of caregivers; 1–30h, 60% of caregivers; ≥30h, 30% of caregivers), interruptions of sleep during the night (number of nights per week), frequency of perceived burden and limitations in personal life due to caregiving (never, rarely-sometimes, often-almost always), and type of caregiver-care receiver relationship (spousal, non-spousal). 

Two independent exposures were used in the multinomial models. The social isolation index was operationalized as a standardized Z-score derived from multiple indicators of social connections, including marital status, number of children, and frequency of social contacts. Each component was standardized and then combined into a single index, with higher scores reflecting higher levels of social isolation, as previously described [23]. For this study, social isolation was categorized into tertiles (low, moderate, high), and also used as a continuous variable in interaction analyses. Loneliness was determined by asking a single-item question “*How often do you feel lonely?*” and was categorized into three reported frequency levels (never, rarely, sometimes/often). These exposures were considered and modelled independently, since they measure different social constructs and are often weakly correlated (Cramér’s V = 0.16 in our dataset).

### Statistical analysis

A combination of multiple correspondence analysis (MCA) and cluster analysis was employed to identify data-driven profiles of informal caregivers. Rather than assuming a simple high/low continuum of QoL, MCA was used to explore the dimensionality of the SF-12 QoL instrument items and uncover underlying relationships between them. The main dimensions explaining the maximum variability of the data were selected and included in a k-means cluster analysis to group individuals based on their QoL dimensions. The CH Index was used to determine the optimal number of clusters, and Jaccard bootstrap coefficients assessed cluster stability.

Descriptive statistics were used to characterize the obtained QoL clusters, with categorical data presented as frequencies and percentages (n, %), and continuous variables as means with standard deviations (SD) or medians with interquartile ranges for non-normally distributed data. Multinomial logistic regression models were employed to study the associations between levels of loneliness and social isolation, and the probability of belonging to a given QoL profile (taking the best QoL profile as the reference). These models were adjusted for several factors derived from a theoretical framework [24] that were available in our data (age, sex, education, caregiver’s own need for care, spousal caregiving, hours of care provided and caregiving burden), and accounted for repeated measurements by using robust standard errors for clustered data. Relative risk ratios (RRR) with 95% confidence intervals (lower and upper bounds, LB, UB) and *p*-values were reported. Analyses were performed using statistical software R, version 4.4.1, and Stata, release 17 (College Station, TX: StataCorp LLC).

## Results

**Fig. 1:**
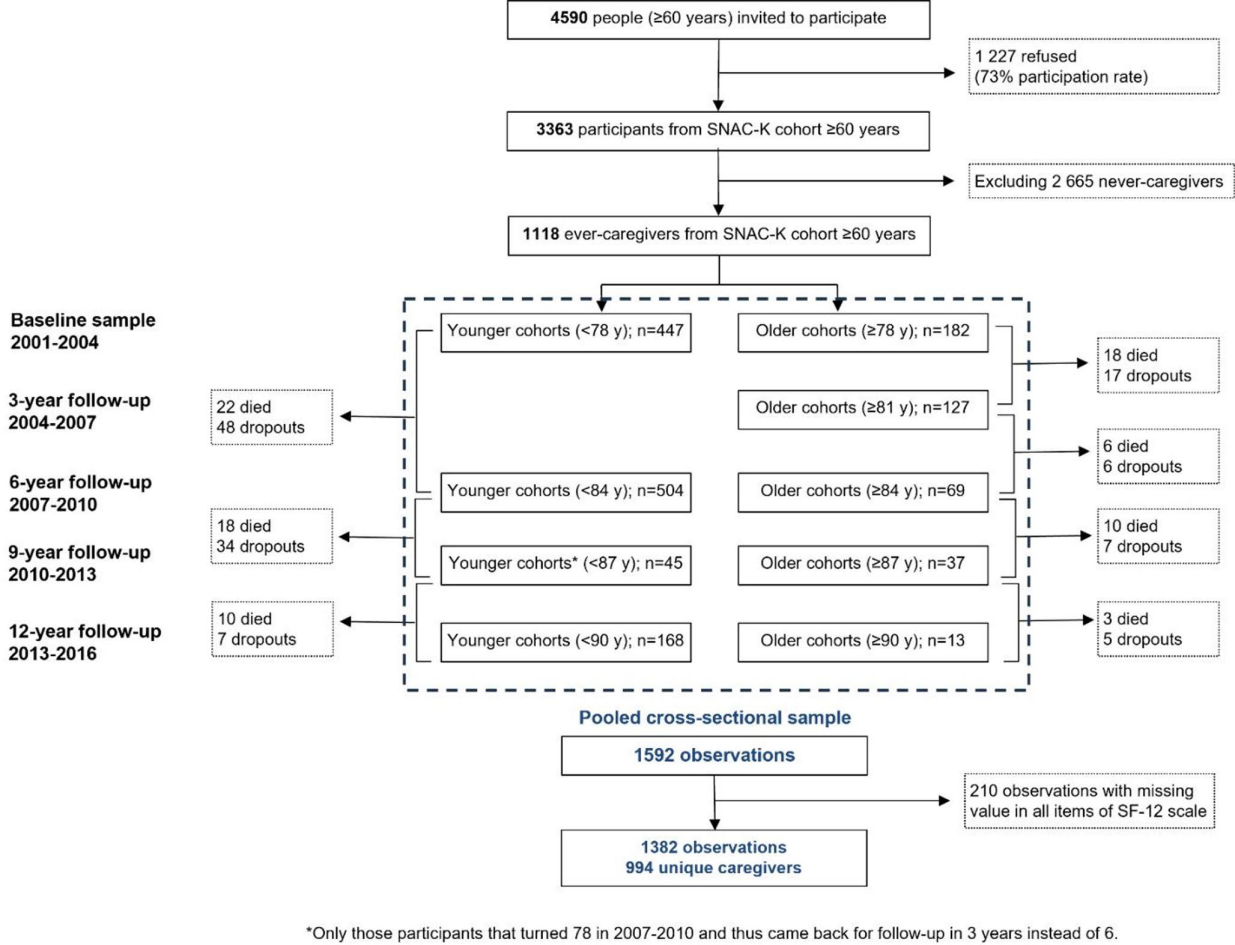
Study population flowchart

A total of 994 unique caregivers were providing care at some point during the study period, leading to a total of 1382 observations. The selection of the study sample is summarized in the study population flowchart (Fig. 1). The mean age of caregivers in SNAC-K was 73 years (SD 8.3) and 66% were female.

**Fig. 2:**
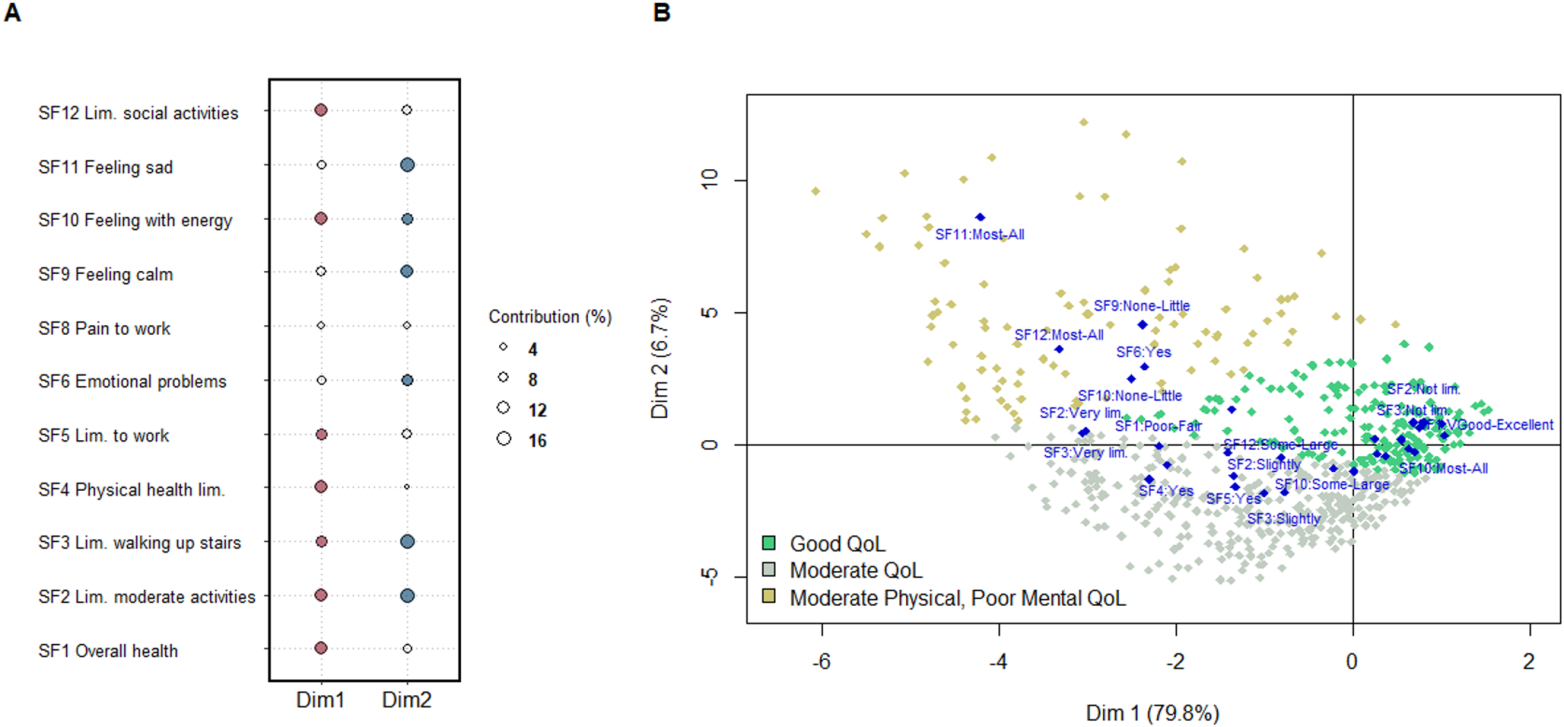
Informal caregivers’ QoL profiles: results from the MCA and cluster analysis. **A**. Contribution of each item to the construction of both MCA dimensions, the larger the dot, the greater the contribution to that dimension. Most meaningful items (i.e., contributions higher than 9%) are plotted in color (red for dimension 1 and blue for dimension 2). **B**. Map display of the SF-12 items with their categories (blue diamonds) and three clusters (green, gray and yellow dots) across the two dimensions of the MCA analysis

The outputs of the MCA and cluster analysis are shown in Fig. 2. The first two dimensions of the MCA explained 85.9% of the data variability, 79.8% and 6.1%, respectively. The contribution of each variable and response options to both dimensions is presented in Fig. 2A. Three clusters—*good QoL* (57.9% of the sample), *moderate QoL* (34.8% of the sample) and *moderate physical*,* poor mental QoL* (7.3% of the sample)—were identified by the k-means method, with a good cluster stability as indicated by the Jaccard bootstrap coefficients: 0.96, 0.97, and 0.93, respectively. The representation of the clusters, together with the QoL items, are shown in Fig. 2B. Differences in each item of the QoL scale are presented in Supplementary Table 2.

A description of the total sample and the three QoL profiles is presented in Table 1. The *moderate physical*,* poor mental QoL* profile was characterized by an overrepresentation of women, university-educated and unmarried persons, with inadequate physical activity and high depression scores, who provided the most intense care (in hours), often to a spouse. They reported the highest limitations to life and feelings of burden due to caregiving.

**Table 1:** Description of the sample and the caregiver QoL profiles

	Total	Good QoL	Moderate QoL	Moderate Physical, Poor Mental QoL
N	1382	800 (57.9%)	481 (34.8%)	101 (7.3%)
*Quality of life*				
QoL total score, *mean (SD)*	36.7 (5.5)	39.8 (3.2)	33.7 (4.1)	25.7 (4.7)
QoL mental component, *mean (SD)*	20.1 (3.6)	21.4 (2.5)	19.7 (2.7)	11.8 (2.7)
QoL physical component, *mean (SD)*	16.6 (2.8)	18.4 (1.2)	14 (2.1)	13.8 (3.5)
*Sociodemographic and lifestyle factors*				
Age*, mean (SD)*	73.3 (8.3)	71.4 (7.6)	76.4 (8.4)	73.8 (9.1)
Sex (female)	911 (65.9%)	508 (63.5%)	330 (68.6%)	73 (72.3%)
*Education level*				
Elementary	130 (9.4%)	65 (8.1%)	58 (12.1%)	7 (6.9%)
High School	647 (46.8%)	357 (44.6%)	245 (50.9%)	45 (44.6%)
University	605 (43.8%)	378 (47.2%)	178 (37%)	49 (48.5%)
*Occupation*				
Manual worker	222 (16.1%)	116 (14.5%)	95 (19.8%)	11 (11%)
Non-manual worker	1157 (83.9%)	683 (85.5%)	385 (80.2%)	89 (89%)
*Civil status*				
Unmarried	196 (14.2%)	108 (13.5%)	61 (12.7%)	27 (26.7%)
Married	775 (56.2%)	477 (59.7%)	253 (52.7%)	45 (44.6%)
Divorced	214 (15.5%)	125 (15.6%)	71 (14.8%)	18 (17.8%)
Widow	195 (14.1%)	89 (11.1%)	95 (19.8%)	11 (10.9%)
*Smoking status*				
Never	619 (45.1%)	362 (45.7%)	211 (44.1%)	46 (45.5%)
Former	591 (43.1%)	337 (42.6%)	218 (45.5%)	36 (35.6%)
Current	162 (11.8%)	93 (11.7%)	50 (10.4%)	19 (18.8%)
*Alcohol consumption*				
Never/Occasionally	320 (23.2%)	139 (17.4%)	144 (30%)	37 (36.6%)
Light-Moderate	801 (58%)	512 (64%)	245 (51%)	44 (43.6%)
Heavy	260 (18.8%)	149 (18.6%)	91 (19%)	20 (19.8%)
*Body Mass Index (BMI)*				
Underweight	14 (1%)	5 (0.6%)	7 (1.5%)	2 (2%)
Normal weight	551 (40%)	346 (43.4%)	158 (33.1%)	47 (47%)
Overweight	619 (45%)	362 (45.4%)	227 (47.5%)	30 (30%)
Obese	192 (14%)	85 (10.7%)	86 (18%)	21 (21%)
*Physical activity levels*				
Inadequate	228 (16.5%)	106 (13.2%)	89 (18.5%)	33 (32.7%)
Health-enhancing	710 (51.4%)	390 (48.8%)	269 (55.9%)	51 (50.5%)
Fitness-enhancing	444 (32.1%)	304 (38%)	123 (25.6%)	17 (16.8%)
*Died during follow up*	206 (14.9%)	75 (9.4%)	104 (21.6%)	27 (26.7%)
*Health status factors*				
HAT score, *mean (SD)*	7.7 (1.3)	8.2 (1.1)	7 (1.4)	7 (1.6)
MADRS score, *mean (SD)*	1.7 (2.8)	1.2 (2.1)	1.8 (2.6)	5.1 (4.8)
Also receives formal/informal care	136 (9.8%)	21 (2.6%)	92 (19.1%)	23 (22.8%)
*Care-related factors*				
Hours of care provided, *mean (SD)*	43.7 (103.5)	43.8 (103.7)	40.3 (95.7)	60.1 (134.6)
Nights with interrupted sleep per week, *mean (SD)*	0.3 (1.7)	0.3 (1.6)	0.3 (1.6)	0.7 (2.8)
*Limitations to live own life*				
No limitations	864 (65.6%)	552 (71.7%)	277 (61.3%)	35 (36.8%)
Slight limitations	259 (19.7%)	134 (17.4%)	102 (22.6%)	23 (24.2%)
Moderate-severe limitations	194 (14.7%)	84 (10.9%)	73 (16.2%)	37 (38.9%)
*Feeling burdened*				
Never	878 (67.7%)	564 (74.7%)	282 (63.2%)	32 (33.3%)
Rarely-sometimes	312 (24.1%)	146 (19.3%)	131 (29.4%)	35 (36.5%)
Often-almost always	107 (8.2%)	45 (6%)	33 (7.4%)	29 (30.2%)
*Spousal care*	276 (20%)	144 (18%)	105 (21.8%)	27 (26.7%)
*Social well-being factors*				
*Social isolation (tertiles)*				
Low	635 (47.4%)	417 (53.9%)	199 (42.5%)	19 (19.6%)
Moderate	451 (33.7%)	239 (30.9%)	168 (36%)	44 (45.4%)
High	253 (18.9%)	118 (15.2%)	101 (21.5%)	34 (35%)
*Ever feel lonely*				
Never	874 (63.6%)	528 (66.2%)	306 (64.2%)	40 (40%)
Rarely	254 (18.5%)	150 (18.8%)	87 (18.2%)	17 (17%)
Sometimes/Often	247 (18%)	120 (15%)	84 (17.6%)	43 (43%)

HAT, Health Assessment Tool, which takes values from 0 to 10 (higher scores indicating better health); MADRS, Montgomery-Åsberg Depression Rating Scale (range 0–60, with higher scores indicating more symptoms).

**Fig. 3:**
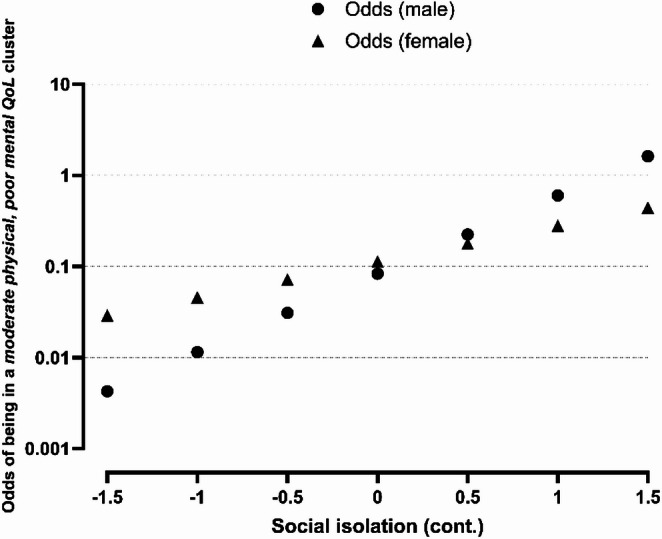
Interaction effect of sex and social isolation against the probability of belonging to the *moderate physical*,* poor mental QoL versus good QoL* profile

Compared with caregivers in the *good QoL* profile, those in the *moderate physical*,* poor mental QoL* profile were 3.2 times (95% CI: 1.6–6.3) more likely to belong to the moderate social isolation tertile and 5.6 times (95% CI: 2.7–11.6) more likely to belong to the high social isolation tertile. They also had a 1.7-times (95% CI: 0.9–3.5) and 4.8-times (95% CI: 2.6–9.0) higher risk of reporting loneliness as rarely or sometimes/often (as opposed to never), respectively, compared to those in the *good QoL* profile (Table 2). No significant associations were seen for the *moderate QoL* vs. *good QoL* profile comparison. We observed a significant interaction between sex and social isolation (*p* = 0.02). As shown in Table 2; Fig. 3, the odds of belonging to the *moderate physical*,* poor mental QoL* profile increased more steeply with higher levels of social isolation among males than among females, indicating a stronger association between social isolation and poorer QoL in men.

**Table 2:** Multinomial logistic regression models depicting the relationship between social isolation and loneliness levels and the probability of belonging to the different QoL profiles. The estimates are adjusted for age, sex, education, receiving care themselves, spousal care, hours of provided care and caregiving burden

	Moderate vs Good QoL					Moderate Phys. Poor Ment. vs Good QoL				
	*RRR*	*SE*	*p*	*LB*	*UB*	*RRR*	*SE*	*p*	*LB*	*UB*
*Social isolation* ^*^										
Low (reference)	–	–	–	–	–	–	–	–	–	–
Moderate	1.00	0.17	0.99	0.71	1.40	**3.22**	**1.11**	**0.01**	**1.64**	**6.32**
High	1.23	0.25	0.31	0.82	1.85	**5.58**	**2.09**	**0.01**	**2.68**	**11.64**
Sex (male vs female)#Social isolation (continuous) interaction^¥^	1.09	0.29	0.35	0.65	1.85	**0.34**	**0.16**	**0.02**	**0.13**	**0.87**
*Loneliness*										
Never (reference)	–	–	–	–	–	–	–	–	–	–
Rarely	0.86	0.16	0.44	0.60	1.25	1.74	0.62	0.13	0.86	3.51
Sometimes/Often	0.95	0.19	0.79	0.64	1.40	**4.81**	**1.53**	**0.01**	**2.58**	**8.99**

^*^The social isolation index, operationalized as a Z-score, consisted of several items related to the quantification of social connections (such as marital status, number of children and frequency of contacts).^22^

^¥^We evaluated interactions between all covariates and the variables representing social isolation and loneliness; however, none of these interactions, except for sex, were found to be statistically significant.

RRR, Relative Risk Ratio; SE, Standard Error; LB, Lower Bound; UB, Upper Bound. Bold estimates indicate statistical significance.

## Discussion

This study offers new insights into the QoL of older informal caregivers in Sweden, highlighting key factors that affect their well-being. We identified three distinct QoL profiles: *good*, *moderate*, and *moderate physical*,* poor mental QoL*. While most caregivers fell into the *good* or *moderate QoL* profiles, some were categorized in the *moderate physical*,* poor mental QoL* profile. While it is well established that loneliness and social isolation adversely affect older adults’ well-being, our study extends this knowledge by showing that, within the caregiving population, these social factors are specifically linked to the mental rather than physical dimensions of QoL. By adopting a data-driven clustering approach, we identified distinct caregiver profiles instead of treating QoL as a single continuum, providing a more nuanced understanding of how well-being dimensions co-occur.

The descriptive analysis of the three QoL profiles in this study highlights the significant heterogeneity among older caregivers, whose own health strongly influences their well-being. Caregivers in the poorest QoL profile (*moderate physical*,* poor mental*), who were more likely to be women, spousal caregivers, and those providing more hours of care, had more depressive symptoms and were frequently in need of care themselves. These findings are not surprising and align with previous literature, which shows that older caregivers often have their own health needs that must be addressed in addition to their caregiving responsibilities [9]. The overrepresentation of women and spousal caregivers in the worst QoL profile further emphasizes the challenges these caregivers face, particularly given their higher caregiving intensity and increased emotional and physical strain [25, 26]. Our findings add to this literature by empirically demonstrating the coexistence of poor physical and mental QoL within a subset of caregivers, reinforcing the need to view caregiver well-being as a multidimensional concept.

Loneliness and social isolation emerged as significant factors associated with the worst QoL profile. Previous research suggests that spousal caregivers are at risk of social isolation because they often feel ashamed of their care recipient’s behaviors or are simply too busy to maintain social connections [27, 28]. Additionally, the caregiving burden can lead to a reduction in leisure time, further exacerbating isolation [29]. We also found that the most significant differences in QoL were between the worst and best profiles, rather than between the middle and best profiles. This suggests that it is primarily the mental component of QoL that is correlated with loneliness and isolation. Caregivers in the *moderate* profile still reported relatively high mental QoL, indicating that they are better able to manage the psychological challenges associated with caregiving. In contrast, caregivers in the worst profile, with poorer mental health, were more vulnerable to experiencing social isolation and, subsequently, the many negative health outcomes it has been linked to.

The observed interaction between sex and social isolation provides an additional novel insight. While women were overrepresented in the poorest QoL group, suggesting higher caregiving intensity, men with similarly high levels of social isolation were more likely than women to belong to this worse profile. This suggests that men may be particularly vulnerable to the psychological consequences of isolation, potentially due to smaller social networks, a greater reliance on spouses for social connection, or less access to emotional support [30, 31]. Conversely, women may be somewhat better able to cope with isolation, possibly through stronger relational or coping resources. These findings highlight that sex not only shapes caregiving roles but also moderates how social isolation translates into mental well-being.

### Implications for policy and research

The findings of this study highlight the need for policies that address the complex challenges faced by older informal caregivers, particularly those providing high-intensity spousal care. Our results show that a subgroup of older caregivers experience low QoL, characterized by poor mental well-being, loneliness, and social isolation, pointing to the need for targeted psychosocial and gender-sensitive interventions. Although Sweden offers support to caregivers, access varies significantly across municipalities, and caregiver engagement with available services remains suboptimal [32]. Furthermore, regular evaluations of these services are lacking, making it difficult to assess their impact or effectiveness.

Further research is essential to investigate the intersectional and longitudinal dynamics of sociodemographic, psychosocial, and caregiving-related factors on caregivers’ QoL. Such studies should aim to uncover pathways through which targeted interventions can effectively alleviate caregiver burden and isolation over time. By integrating these findings into policies, we can ensure that the diverse experiences and needs of caregivers are accounted for.

## Strengths and limitations

This study has several key strengths. The use of robust statistical methods, including multiple correspondence and cluster analyses, allowed for a detailed identification of caregiver profiles based on QoL measures. Additionally, the study benefits from rich, reliable data from an ongoing cohort study. A major strength is the focus on older informal caregivers, an often-overlooked group, offering valuable insights into their challenges.

However, there are several limitations. The cross-sectional design limits the ability to establish temporality, making it unclear whether worse QoL leads to or results from loneliness and social isolation. Most likely, this association is bidirectional. The lack of information about care receivers (e.g., their health status or care needs) limits understanding of the caregiver/care receiver dynamics. Additionally, our dataset included 11 of the 12 items from the SF-12 tool, with item 7 not available in the original data collection. Although this omission slightly reduces the completeness of the standard SF-12 instrument, its impact on our findings is likely minimal. First, summation of raw item scores is a recognized method for operationalizing SF-12 constructs [19]. Second, because each SF-12 item was analyzed separately in the MCA, rather than being aggregated into summary scores, the absence of a single item does not distort the clustering structure or bias the identification of QoL profiles. Finally, the cohort for this study was drawn from Kungsholmen, a central island in Stockholm, Sweden, characterized by a predominantly white and middle-to-high-income population. This limits the generalizability of the results to more ethnically diverse and/or low-income settings.

## Conclusion

This study highlights the heterogeneity in QoL of older informal caregivers in Sweden, revealing three distinct profiles: *good*, *moderate*, and *moderate physical*,* poor mental QoL*. While most caregivers reported good or moderate QoL, a notable subset experienced poor mental health, and belonging to the latter group was strongly associated with loneliness and social isolation. Women, spousal caregivers, and those providing intensive care were also overrepresented in this group, underscoring the substantial emotional and physical strain they face. The findings emphasize the critical role of social well-being in maintaining caregivers’ QoL, with sex-specific differences highlighting men’s heightened vulnerability to isolation. Tailored support services, equitable access to resources, and regular evaluations of caregiver interventions are necessary to address these challenges. Future research should focus on the longitudinal and intersectional factors influencing caregivers’ QoL to guide evidence-based policies that support their well-being and recognize their vital societal contributions.

## Supplementary Information

Below is the link to the electronic supplementary material.

Supplementary Material 1

## Data Availability

The data originate from the SNAC-K project, an ongoing population-based study in Sweden (http://www.snac-k.se/). Researchers can request access to the data by applying to the SNAC-K data management committee. Applications should be submitted through the website.
