# Supplementary material for: Quality of life among older informal caregivers in Sweden: the role of loneliness and social isolation
Source: Qual Life Res. 2026 Feb 1;35(3):52. doi: 10.1007/s11136-025-04156-x (PMC12862014; doi:10.1007/s11136-025-04156-x)
Supplement: Supplementary file 1 — Supplementary Material 1 [file 11136_2025_4156_MOESM1_ESM.docx]

**Quality of life among older informal caregivers in Sweden: The role of loneliness and social isolation**

**SUPPLEMENTARY MATERIALS**

**Supplementary Table 1.** STROBE Statement—Checklist of items that should be included in reports of cross-sectional studies.

|  | Item No | Recommendation | Page No |
| --- | --- | --- | --- |
| Title and abstract | 1 | (a) Indicate the study’s design with a commonly used term in the title or the abstract | 2 |
|  |  | (b) Provide in the abstract an informative and balanced summary of what was done and what was found | 2 |
| Introduction | | | |
| Background/rationale | 2 | Explain the scientific background and rationale for the investigation being reported | 4 |
| Objectives | 3 | State specific objectives, including any prespecified hypotheses | 4 |
| Methods | | | |
| Study design | 4 | Present key elements of study design early in the paper | 4 |
| Setting | 5 | Describe the setting, locations, and relevant dates, including periods of recruitment, exposure, follow-up, and data collection | 4 |
| Participants | 6 | (a) Give the eligibility criteria, and the sources and methods of selection of participants | 4 |
| Variables | 7 | Clearly define all outcomes, exposures, predictors, potential confounders, and effect modifiers. Give diagnostic criteria, if applicable | 5 |
| Data sources/ measurement | 8* | For each variable of interest, give sources of data and details of methods of assessment (measurement). Describe comparability of assessment methods if there is more than one group | 5 |
| Bias | 9 | Describe any efforts to address potential sources of bias | 5 |
| Study size | 10 | Explain how the study size was arrived at | NA |
| Quantitative variables | 11 | Explain how quantitative variables were handled in the analyses. If applicable, describe which groupings were chosen and why | 5 |
| Statistical methods | 12 | (a) Describe all statistical methods, including those used to control for confounding | 5 |
|  |  | (b) Describe any methods used to examine subgroups and interactions | 5 |
|  |  | (c) Explain how missing data were addressed | NA |
|  |  | (d) If applicable, describe analytical methods taking account of sampling strategy | NA |
|  |  | (e) Describe any sensitivity analyses | NA |
| Results | | | |
| Participants | 13* | (a) Report numbers of individuals at each stage of study—eg numbers potentially eligible, examined for eligibility, confirmed eligible, included in the study, completing follow-up, and analysed | 6, Fig1 |
|  |  | (b) Give reasons for non-participation at each stage | Fig1 |
|  |  | (c) Consider use of a flow diagram | Fig1 |
| Descriptive data | 14* | (a) Give characteristics of study participants (eg demographic, clinical, social) and information on exposures and potential confounders | Table1 |
|  |  | (b) Indicate number of participants with missing data for each variable of interest | NA |
| Outcome data | 15* | Report numbers of outcome events or summary measures | Table2 |
| Main results | 16 | (a) Give unadjusted estimates and, if applicable, confounder-adjusted estimates and their precision (eg, 95% confidence interval). Make clear which confounders were adjusted for and why they were included | 6 |
|  |  | (b) Report category boundaries when continuous variables were categorized | 6 |
|  |  | (c) If relevant, consider translating estimates of relative risk into absolute risk for a meaningful time period | NA |
| Other analyses | 17 | Report other analyses done—eg analyses of subgroups and interactions, and sensitivity analyses | NA |
| Discussion | | | |
| Key results | 18 | Summarise key results with reference to study objectives | 6 |
| Limitations | 19 | Discuss limitations of the study, taking into account sources of potential bias or imprecision. Discuss both direction and magnitude of any potential bias | 7 |
| Interpretation | 20 | Give a cautious overall interpretation of results considering objectives, limitations, multiplicity of analyses, results from similar studies, and other relevant evidence | 6,7 |
| Generalisability | 21 | Discuss the generalisability (external validity) of the study results | 8 |
| Other information | | | |
| Funding | 22 | Give the source of funding and the role of the funders for the present study and, if applicable, for the original study on which the present article is based | 11 |

*Give information separately for exposed and unexposed groups.

**Supplementary Table 2.** SF-12 item distribution between QoL profiles.

|  | **Total** | **Good QoL** | **Moderate QoL** | **Moderate Physical, Poor Mental QoL** |
| --- | --- | --- | --- | --- |
| **N** | **1382** | **800 (57.9%)** | **481 (34.8%)** | **101 (7.3%)** |
| **Items of SF12 QoL scale** |  |  |  |  |
| Overall health |  |  |  |  |
| Poor/fair | 285 (20.9%) | 38 (4.8%) | 179 (37.6%) | 68 (68.7%) |
| Good | 469 (34.3%) | 221 (27.9%) | 232 (48.7%) | 16 (16.2%) |
| Very good/Excellent | 612 (44.8%) | 532 (67.3%) | 65 (13.7%) | 15 (15.2%) |
| Limited moderate activities |  |  |  |  |
| Not limited | 866 (63.1%) | 754 (94.8%) | 72 (15%) | 40 (40.8%) |
| Slightly limited | 425 (31%) | 40 (5%) | 356 (74.2%) | 29 (29.6%) |
| Very limited | 82 (6%) | 1 (0.1%) | 52 (10.8%) | 29 (29.6%) |
| Limited ability to climb stairs |  |  |  |  |
| Not limited | 859 (62.4%) | 720 (90.6%) | 92 (19.1%) | 47 (47%) |
| Slightly limited | 434 (31.5%) | 74 (9.3%) | 338 (70.3%) | 22 (22%) |
| Very limited | 83 (6%) | 1 (0.1%) | 51 (10.6%) | 31 (31%) |
| Accomplished less than desired due to physical health |  |  |  |  |
| No | 1089 (79.3%) | 763 (96.2%) | 280 (58.3%) | 46 (46%) |
| Yes | 284 (20.7%) | 30 (3.8%) | 200 (41.7%) | 54 (54%) |
| Limited work/other activities due to physical health |  |  |  |  |
| No | 879 (64.5%) | 712 (90.1%) | 128 (27%) | 39 (39.8%) |
| Yes | 483 (35.5%) | 78 (9.9%) | 346 (73%) | 59 (60.2%) |
| Accomplished less than desired due to emotional problems |  |  |  |  |
| No | 1230 (89.7%) | 756 (95.3%) | 446 (92.9%) | 28 (28.3%) |
| Yes | 142 (10.3%) | 37 (4.7%) | 34 (7.1%) | 71 (71.7%) |
| Pain interfered with work |  |  |  |  |
| Extremely/Quite a bit | 50 (3.7%) | 3 (0.4%) | 38 (8%) | 9 (9.2%) |
| Moderately | 128 (9.4%) | 20 (2.5%) | 93 (19.6%) | 15 (15.3%) |
| Not at all/A little | 1189 (87%) | 772 (97.1%) | 343 (72.4%) | 74 (75.5%) |
| Felt calm and peaceful |  |  |  |  |
| Little/None of the time | 98 (7.1%) | 23 (2.9%) | 8 (1.7%) | 67 (67%) |
| Good bit/Some of the time | 437 (31.9%) | 195 (24.6%) | 213 (44.5%) | 29 (29%) |
| All/Most of the time | 836 (61%) | 574 (72.5%) | 258 (53.9%) | 4 (4%) |
| Felt full of energy |  |  |  |  |
| Little/None of the time | 163 (11.9%) | 30 (3.8%) | 53 (11.1%) | 80 (80%) |
| Good bit/Some of the time | 671 (49%) | 326 (41.2%) | 328 (68.6%) | 17 (17%) |
| All/Most of the time | 536 (39.1%) | 436 (55.1%) | 97 (20.3%) | 3 (3%) |
| Felt sad/blue |  |  |  |  |
| Little/None of the time | 23 (1.7%) | 0 (0%) | 0 (0%) | 23 (22.8%) |
| Good bit/Some of the time | 233 (17%) | 99 (12.5%) | 71 (14.8%) | 63 (62.4%) |
| All/Most of the time | 1115 (81.3%) | 692 (87.5%) | 408 (85.2%) | 15 (14.9%) |
| Limited social activities due to physical or emotional problems |  |  |  |  |
| Little/None of the time | 68 (5%) | 5 (0.6%) | 17 (3.6%) | 46 (46%) |
| Good bit/Some of the time | 292 (21.4%) | 85 (10.8%) | 163 (34.4%) | 44 (44%) |
| All/Most of the time | 1002 (73.6%) | 698 (88.6%) | 294 (62%) | 10 (10%) |

There were 67 missing values for the total score and the number of missing values for each item ranged from 6 to 20.
